# The relationship between missed nursing care and job burnout in intensive care units

**DOI:** 10.25122/jml-2024-0347

**Published:** 2025-11

**Authors:** Shahin Shabani, Neda Sheikhzakaryaee, Shahnaz Salawati Ghasemi, Arian Azadnia

**Affiliations:** 1 Department of Nursing and Midwifery, Kurdistan University of Medical Sciences, Sanandaj, Iran; 2 Department of Epidemiology & Biostatistics, School of Medicine, Kurdistan University of Medical Sciences, Sanandaj, Iran

**Keywords:** missed nursing care, job burnout, nurse, intensive care unit

## Abstract

Providing quality nursing care in intensive care units is a crucial component of hospital services. Conversely, the stress and workload in these environments put nurses at risk of job burnout. This study aimed to examine the relationship between missed nursing care and job burnout among nurses in intensive care units. This cross-sectional study used census sampling to recruit 200 nurses working in the intensive care units of teaching hospitals at Kurdistan University of Medical Sciences in Iran. The data were collected using demographic information forms, the Kalisch Missed Nursing Care Questionnaire, and the Persian version of the Maslach Burnout Inventory (MBI). Descriptive and analytical analyses were performed using SPSS software version 24. In this study, the mean score of missed nursing care was 35.29 ± 9.43. Additionally, a moderate level of job burnout among nurses was found, with a mean score of 42.54 ± 17.22. The results indicated a significant relationship between missed nursing care and nurses' gender and economic status (*P* < 0.05). Furthermore, missed nursing care was significantly related to job burnout (positive correlation) and its dimensions, including depersonalization and personal accomplishment (*P* < 0.05). Based on these findings, to enhance the quality of healthcare services in intensive care units, measures should be taken to reduce missed nursing care and job burnout among nurses.

## Introduction

Recent research has shown that missed nursing care is a highly prevalent phenomenon in acute care settings [1]. This issue has been recognized as a critical concept in nursing within acute care facilities worldwide [2] and is considered one of the forms of healthcare deficiency on a global scale [3]. Missed nursing care refers to the neglect of both minor and major aspects of patient care that are either forgotten or delayed [2], partially completed, or not completed at all. In another definition, it involves essential care necessary for optimal, rapid patient recovery, but is omitted or not performed [3]. Moreover, missed nursing care is not only a type of nursing error that can affect patient safety but also a form of neglect that violates patients' rights and jeopardizes their rehabilitation and recovery [2].

Nursing care is particularly important due to its direct impact on patient recovery and health [4]. It can also serve as a benchmark for comparing performance across systems and organizations [5]. Several factors, such as understaffing, poor teamwork and safety, and work-related stress, contribute to missed care [6]. Emotional exhaustion among nurses is one such factor that leads to diminished care quality [7]. Indeed, job burnout and occupational stress among healthcare workers pose threats to patient safety and the quality of care. Job burnout among healthcare workers is an independent predictor of adverse events, healthcare-associated infections, and medical errors, and is associated with poor patient satisfaction [8].

Among the job-related consequences of burnout are withdrawal from work, absenteeism, changes in job performance, and decreased activity and effectiveness at work for those who remain in their positions [9]. Therefore, job burnout and job dissatisfaction not only lead to staff turnover but also negatively affect the quality of care [10]. When a nurse experiences burnout, patients are deprived of adequate attention and care, and their individual human rights are called into question. A nurse suffering from burnout, due to depersonalization, responds negatively to care recipients [11]. The evidence suggests that nurse burnout affects patient recovery more than any other factor [12].

Studies indicate that 23 to 43% of intensive care unit (ICU) nurses worldwide suffer from job burnout [13], characterized by high levels of emotional exhaustion (EE) and depersonalization, and low levels of personal accomplishment (PA) [14]. Providing care in the ICU requires nurses to regularly handle the most critical cases [14], making ICU work more stressful and impacting the quality of care they provide [15]. Recent evidence indicates that insufficient, delayed, or omitted nursing care is widespread among nurses [5]. In fact, nurses' ability to provide care and fulfill their roles is greatly influenced by the work environment in which they serve [10].

Several studies have shown that missed nursing care is prevalent in hospitals in the United States and Europe [16,17]. In a study conducted in the United States, the prevalence of missed nursing care was reported to be between 10 to 27% [18]. A review of the literature indicates that this issue is highly context-dependent, with frequency and type varying across hospitals within the same country and even across units within the same hospital [19]. In Iran, the rate of missed nursing care has been reported to be greater than that in other parts of the world [20]. For instance, a recent Iranian study revealed that 72.1% of nurses missed at least one nursing care activity during their last shift [21].

Studies on missed nursing care highlight the greater importance of quality and comprehensive nursing care in ICUs than in other departments. Therefore, assessing factors such as job burnout, which may be related to missed nursing care in ICUs, is crucial. However, few studies have specifically examined the relationship between missed nursing care and job burnout, and none have been conducted recently, particularly in Iran or within ICU settings. Thus, this study aimed to determine the relationship between missed nursing care and job burnout among nurses in intensive care units.

## Material and Methods

This cross-sectional study included nurses working in the intensive care units of teaching hospitals at Kurdistan University of Medical Sciences in Iran in 2023. The research sample was selected from the research population based on the following inclusion criteria: having rotating shift duties, at least 6 months of clinical experience in an intensive care unit, willingness to participate in the study, and holding a bachelor's degree or higher. The sampling method used was convenience sampling. The exclusion criterion was incomplete completion of the questionnaires.

The research environment for this study comprised 11 intensive care units in Kosar, Towhid, and Besat educational hospitals affiliated with Kurdistan University of Medical Sciences. To determine the sample size, a census method was used, and all nurses working in the intensive care units of these teaching hospitals in 2023 were included in the study. It is worth mentioning that the total number of nurses working in the intensive care units of Kurdistan's teaching hospitals was 253. Of these, 23 did not meet the inclusion criteria, and out of the remaining 230, 200 completed the questionnaires.

In this study, the data collection tools included a demographic information form for nurses, the Kalisch Missed Nursing Care Questionnaire, and the Persian version of Maslach Burnout Inventory (MBI). The demographic information form comprised questions related to age, gender, marital status, education, employment status, economic status, place of residence, place of employment, length of employment in ICUs, length of employment overall, number of shifts per month, type of shift, total working hours per month, and the amount of overtime per month.

In the present study, the Kalisch Missed Nursing Care Questionnaire was used to assess missed nursing care. This questionnaire was developed by Kalisch in 2006 and was psychometrically evaluated by the same researcher in 2009. It consists of 24 items, including patient movement, turning, assessment, education, discharge planning, and medication administration. Each item is rated on a four-point Likert scale ranging from 1 to 4. Higher scores on this questionnaire indicate a higher level of missed nursing care, and vice versa. The total score on this questionnaire ranges from 24 to 96 [20]. Its validity in Iran was established by Khajavi and colleagues in 2019, with a reported coefficient of 0.91 [20]. In a subsequent study by Ebadi and colleagues, the reliability of the instrument, assessed using Cronbach’s alpha, was 0.88 [2]. In the present study, face validity was evaluated through feedback from 10 faculty members with expertise in nursing care, and their suggestions were incorporated into the final version of the questionnaire. Reliability was then assessed using Cronbach’s alpha.

The Persian version of the Maslach Burnout Inventory (MBI) was used to measure job burnout among nurses. This questionnaire consists of 22 items designed to assess three dimensions of job burnout: emotional exhaustion (9 items), depersonalization (5 items), and reduced personal accomplishment (8 items). High scores in the dimensions of emotional exhaustion and depersonalization, along with low scores in personal accomplishment, indicate a high level of job burnout. The scores for each dimension are considered separately and are not combined into a single total score. In the study by Azimilolaty and colleagues, the reliability coefficients for the dimensions of emotional exhaustion, depersonalization, and personal accomplishment were 0.90, 0.79, and 0.71, respectively [22]. In the study by Rasoul Aghadavoud, the Cronbach's alpha coefficient for the MBI was 0.852 [23].

In this study, data analysis was conducted using SPSS version 24 software. Descriptive analysis of the data was performed using frequency tests, means, and standard deviation calculations. To explore the relationships between variables, various statistical tests, including *t*-tests, Mann-Whitney U tests, ANOVA, and Kruskal-Wallis tests, were used. Furthermore, linear regression analysis was conducted to evaluate correlations between variables. The significance level was set at 5%.

## Results

The results of this study, conducted among 200 nurses working in the intensive care units of educational hospitals affiliated with Kurdistan University of Medical Sciences, indicated that the participants' mean age was 32.05 ± 6.09 years. Most of the nurses were women (58.0%), married (54.0%), held a bachelor's degree (94.5%), were officially employed (49.0%), had a moderate economic status (65.5%), and resided in urban areas (99.0%). Nearly half of the participants worked at Kowsar Hospital (48.5%). Furthermore, the mean duration of employment in special care units was 21.5 ± 4.41 months, while the mean overall work experience was 17.04 ± 20.13 months. In addition, the nurses worked an average of 20.00 ± 15.52 shifts per month, with a mean total of 187.06 ± 28.56 working hours (Table 1).

**Table 1:** Demographic characteristics of nurses employed in the intensive care department of teaching hospitals, Sanandaj, Iran (*n* = 200)

Demographic characteristics		Mean ± (SD) or Number (%)
**Age**		32.05 ± 6.09
**Gender**	Men	84 (42.0%)
	Women	116 (58.0%)
**Marital status**	Married	109 (54.5%)
	Single	85 (42.5%)
	Divorced	5 (2.5%)
	Widowed/ Widower	1 (0.5%)
**Level of education**	Associate's degree	1 (0.5%)
	Bachelor's degree	189 (94.5%)
	Master's degree	10 (5.0%)
**Employment status**	Employed by a company	10 (5.0%)
	Contractual	52 (26.0%)
	Fixed-term contract	35 (17.5%)
	Official	98 (49.0%)
	Service commitment period	5 (2.5%)
**Economic status**	Weak	43 (21.5%)
	Moderate	131 (65.5%)
	Good	26 (13.0%)
**Place of residence**	City	198 (99.0%)
	Village	2 (1.0%)
**Workplace hospital**	Kawsar Hospital	97 (48.5%)
	Tohid Hospital	87 (43.5%)
	Be'sat Hospital	16 (8.0%)
**Duration of employment in the ICU**		5.21 ± 4.41
**Duration of nurses' employment**		17.04 ± 120.31
**Number of nursing shifts**		20.00 ± 15.52
**The working hours of nurses**		187.06 ± 28.56
**Work shift type**	Fixed shift	39 (19.5%)
	Rotating shift	161 (80.5%)
**Overtime hours per month**	100>	190 (95.0%)
	100<	10 (5.0%)

The results of the present study indicated that among nurses working in the special care units of educational hospitals affiliated with Kurdistan University of Medical Sciences, the mean score for missed nursing care was 29.35 ± 43.9. The mean scores for emotional exhaustion, depersonalization, and personal accomplishment were 25.14 ± 11.12, 9.4 ± 56.5, and 19.24 ± 5.9, respectively. The overall occupational burnout score of nurses was 54.42 ± 22.17 (Table 2).

**Table 2:** The distribution of scores for missed nursing care, nurse burnout, and dimensions of nurse burnout (*n* = 200)

Scores	Mean ± (SD)
**Missed nursing care**	35.29 ± 9.43
**Emotional exhaustion**	14.25 ± 12.11
**Depersonalization**	4.05 ± 5.56
**Personal accomplishment**	24.19 ± 9.05
**Nurse burnout**	42.54 ± 17.22

The results of the present study, based on the Mann–Whitney test, revealed a significant relationship between missed nursing care scores and several demographic variables, including gender (*P* = 0.024), economic status (*P* = 0.0001), and the hospital of employment (*P* = 0.0001). Furthermore, according to the Spearman correlation test, there were significant positive correlations between missed nursing care and the dimensions of emotional exhaustion (P = 0.0001), depersonalization (*P* = 0.0001), personal accomplishment (*P* = 0.0001), as well as the overall occupational burnout score (*P* = 0.045) among nurses working in special care units (Table 3).

**Table 3:** Scores of missed nursing care and its association with demographic characteristics, nurse burnout, and its dimensions, Sanandaj, Iran (*n* = 200)

Missed nursing care score Demographic characteristics		Mean ± (SD) or frequency (average rank)	Spearman’s rho	*P* value
**Scores of missed nursing care**		35.29 ± 9.43		
**Age**		32.05 ± 6.09	- 0.032	0.654
**Gender**	Men	84 (111.36)		0.024
	Women	116 (92.63)		
**Marital status**	Married	109 (109.07)		0.091
	Single	85 (88.57)		
	Divorced	5 (110.30)		
	Widowed/ Widower	1 (131.50)		
**Level of education**	Associate's degree	1 (141.50)		0.620
	Bachelor's degree	189 (99.66)		
	Master's degree	10 (112.25)		
**Employment status**	Employed by a company	10 (102.70)		0.320
	Contractual	52 (86.87)		
	Fixed-term contract	35 (112.24)		
	Official	98 (102.75)		
	Service commitment period	5 (111.60)		
**Economic status**	Weak	43 (120.74)		0.0001
	Moderate	131 (107.43)		
	Good	26 (32.10)		
**Place of residence**	City	198 (101.01)		0.246
	Village	2 (50.25)		
**Workplace hospital**	Kawsar Hospital	97 (121.19)		0.0001
	Tohid Hospital	87 (80.53)		
	Besat Hospital	16 (83.69)		
**Work shift type**	Fixed shift	39 (107.05)		0.430
	Rotating shift	161 (98.91)		
**Overtime hours per month**	100>	190 (100.94)		0.639
	100<	10 (92.15)		
**Duration of employment in the ICU**		5.21 ± 4.41	- 0.126	0.075
**Duration of nurses' employment**		17.04 ± 120.31	- 0.030	0.672
**Number of nursing shifts**		15.52 ± 20.00	0.089	0.212
**The working hours of nurses**		187.06 ± 28.56	0.077	0.280
**Emotional exhaustion**		14.25 ± 12.11	0.380	0.000
**Depersonalization**		4.05 ± 5.56	0.318	0.0001
**Personal accomplishment**		24.19 ± 9.05	-0.349	0.0001
**Nurse burnout**		42.54 ± 17.22	0.142	0.045

The results of the present study, based on linear regression analysis, demonstrated a significant relationship between forgotten nursing care and sex (*P* = 0.037) as well as economic status (*P* = 0.0001; Table 4).

**Table 4:** Regression analysis of the relationship between missed nursing care and demographic variables among nurses working in special care units

Variable	B	S. E	Sig	Collinearity	
				Tol	VIF
**Intercept**	-	13.619	0.000	-	-
**Age**	-0.040	0.186	0.740	0.292	3.421
**Gender**	-0.148	1.339	0.037	0.854	1.171
**Marital status**	0.086	1.154	0.225	0.849	1.178
**Education**	0.024	2.799	0.731	0.898	1.113
**Employment status**	0.090	0.674	0.213	0.808	1.238
**Economic status**	-0.331	1.154	0.000	0.829	1.207
**Place of residence**	-0.004	6.298	0.957	0.949	1.053
**Workplace hospital**	-0.133	1.122	0.080	0.738	1.354
**Duration of employment in the ICU**	-0.069	0.221	0.505	0.392	2.550
**Duration of nurses' employment**	0.010	0.005	0.887	0.909	1.100
**Number of nursing shifts**	0.098	0.040	0.140	0.963	1.039
**Work shift type**	-0.013	2.053	0.885	0.564	1.775
**The working hours of nurses**	-0.107	0.026	0.171	0.969	1.437
**Overtime hours per month**	0.022	2.909	0.750	0.927	1.078

## Discussion

The present study aimed to examine the relationship between missed nursing care and job burnout among nurses working in the special care units of educational hospitals affiliated with Kurdistan University of Medical Sciences in 2023. A total of 200 nurses participated in the study. The findings indicated that the overall level of job burnout among the participants was moderate, with moderate emotional exhaustion, low depersonalization, and low personal accomplishment scores. These results are consistent with those reported by Rastjoo and Zandvanian, who also found a moderate overall level of job burnout among nurses, with moderate emotional exhaustion and depersonalization, and low personal accomplishment score [24]. Similarly, Mudallal *et al*., in a study of 407 Jordanian nurses, demonstrated high levels of job burnout in the emotional exhaustion and depersonalization domains, and a moderate level in personal accomplishment [25]. In a study by Khaneghai *et al*., 84% of nurses experienced job burnout [26]. However, a systematic review and meta-analysis by Isfahani *et al*. reported a 25% prevalence of job burnout among Iranian nurses [11]. Furthermore, a systematic review and meta-analysis by Woo *et al*. on the global prevalence of job burnout symptoms among nurses found an overall prevalence of 11.23%. This study included 113 studies for the systematic review and 61 for the meta-analysis, comprising 45,539 nurses from 49 countries across various specialties [27]. Based on these findings, it may be concluded that the prevalence of job burnout among nurses in the special care units of educational hospitals affiliated with Kurdistan University of Medical Sciences is higher than global statistics indicate. Several factors may contribute to this issue, including long working hours, excessive overtime, insufficient staffing relative to workload, frequent fluctuations in demand and response to medical and nursing care, psychological pressures, and stress waves resulting from sudden and critical patient conditions, as well as unexpected critical events such as accidents and physical injuries. Moreover, the imbalance between workload and material and spiritual rewards is one factor that may contribute to nurse burnout in these environments. Therefore, managerial and executive measures to reduce workload pressure, increase staffing levels, establish rest and psychological support programs, and strengthen support systems can help improve nurses' job burnout in these units. Additionally, some of these differences may be attributed to environmental, cultural, and organizational factors associated with each work environment, which require further investigation and analysis.

The results of the present study indicated that the average score of missed nursing care in the special care units of educational hospitals affiliated with Kurdistan University of Medical Sciences was 43.9 ± 29.35. This was in contrast with the findings of Mohammadi *et al*., who reported an average missed nursing care score of 20.24 ± 18.55 in COVID-19 special care units in Kurdistan [28], which was higher than the level observed in the current study. The higher rate of missed nursing care in Mohammadi *et al*.'s study [28] may be attributed to the timing of the study during the COVID-19 pandemic and the impact of the coronavirus crisis on missed nursing care. Additionally, the results of Khajavi *et al*.'s study in Kerman in 2019 showed an average missed nursing care score of 41.7 ± 28.32 [20], which was close to and consistent with the findings of the present study. This contrasts with a study by Hessels *et al*. conducted in the United States, which reported a prevalence of missed nursing care of 10% to 27% [18]. In another study by Blackman *et al*., the rate of missed nursing care was reported as 34% [29], which is consistent with the results of the present study. However, the rate of missed nursing care in the study by Labrague *et al*. in the Philippines was very low at 50.0 ± 35.1 [30]. They predicted that the low rate of missed care was due to inadequate patient surveillance, as well as the most commonly missed nursing activity, hospital and staffing resource levels, and patient safety culture [30].

The present study demonstrated a significant relationship between the score for missed nursing care and nurses' gender in special care units. This finding was consistent with the study by Chegini *et al*. [21]. It should be noted that, in the current study, the frequency of missed nursing care scores was higher among women than among men, whereas Chegini *et al*. reported a higher risk of missed care among men [21]. Most of the reviewed studies did not observe a significant relationship between missed nursing care scores and gender. For instance, studies by Mohammadi *et al*. [28], Ebadi *et al*. [2], and Labrague *et al*. in the Philippines [30] did not report any significant correlation between the average score of missed nursing care and gender. However, considering the results of the present study and corroborating studies, it can be suggested that gender is an important influencing factor on missed nursing care. The finding supports the notion that women are more likely than men to forget nursing care tasks, which may be attributed to childcare responsibilities and family issues that can influence female nurses' work behavior. Therefore, addressing the needs of this group of caregivers is crucial. The mentioned finding can guide policymakers in addressing the issues faced by female nurses and paying more attention to the reasons for missed nursing care among women.

The current study indicated a significant relationship between the score for missed nursing care and nurses' economic status, with individuals of higher economic status having fewer instances of missed nursing care. This finding contrasts with many studies that have not assessed the impact of this factor on missed nursing care, such as those by Ebadi *et al*. [2], Mohammadi *et al*. [28], and others. Based on this finding, it can be argued that improving nurses' economic status may reduce missed nursing care and ultimately improve the quality of nursing care. Therefore, policymakers and health system decision-makers need to prioritize improving nurses' economic status.

Furthermore, the study results showed a statistically significant correlation between the missed nursing care score and the level of nursing burnout in special care units. This finding is consistent with the study by White *et al*. [8], which also demonstrated a significant correlation between missed nursing care and burnout, with nurses experiencing burnout being 4.97 times more likely to have missed nursing care. Additionally, Nantsupawat *et al*. showed that each unit increase in burnout among nurses was associated with a 1.61 times higher likelihood of missed nursing care [31]. Moreover, Abdulrahim Ibrahim and El-Wekil demonstrated a positive and significant relationship between burnout dimensions and missed nursing care in Egypt [32]. On the other hand, the growing phenomenon of burnout syndrome is accompanied by changes in the workplace and increasing professional demands. Work-related stress can lead to dissatisfaction, chronic fatigue, and emotional exhaustion [8]. Therefore, improving management and supervision systems for nursing care to reduce missed care and, consequently, burnout is essential. Strengthening management and supervision systems, providing stress management skills training, and promoting collaboration and interaction can be effective in reducing burnout and missed nursing care.

This study demonstrated a significant correlation between the score for missed nursing care and the dimensions of job burnout — emotional exhaustion, depersonalization, and personal accomplishment — among nurses. This finding is consistent with the study by Abdulrahim Ibrahim and El-Wekil in Egypt, which also showed a significant positive relationship between dimensions of job burnout and missed nursing care [32]. Similarly, Liu *et al*. in China also found this association [33]. Moreover, the experiences of nurses in the study by Harvey *et al*. indicated a relationship with jeopardizing care, professional incongruence, emotional exhaustion, and depersonalization [34].

Therefore, given the association between missed nursing care and depersonalization among nurses, addressing this issue requires developing communication skills and enhancing nurses' personalities. Solutions such as personality development programs and providing psychological counseling can help improve the situation and reduce care omissions.

Based on these findings, it can be suggested that dimensions of job burnout —emotional exhaustion, depersonalization, and personal accomplishment — among nurses may influence their ability to provide care, thereby reducing care omissions. Additionally, organizing courses and workshops to enhance personal accomplishment skills and reduce job burnout and emotional exhaustion can improve nurses' competence and care provision. Furthermore, investigating environmental factors in special care units and creating conducive environmental conditions can help improve nurses' performance in providing care.

Future research is recommended to investigate the influence of social and family factors on neglected nursing care and nurses’ overall performance. Additionally, studies should examine the impact of work environment variables, such as work shifts, job pressures, and stress management, on forgotten nursing care and job burnout among nurses. These investigations could provide deeper insights into the multifaceted causes of care omission and help develop targeted interventions to enhance nurses’ well-being and the quality of patient care.

## Conclusion

The findings of the present study indicate a moderate level of missed nursing care and job burnout among nurses in the special care units of teaching hospitals affiliated with Kurdistan University of Medical Sciences. Despite advances in hospital technology, the implementation of accreditation standards, and overall improvements in healthcare systems, nurses in special care units still experience job burnout and may forget to perform certain care tasks. Therefore, measures should be taken to reduce burnout and address nurses' needs in these units to improve the quality of healthcare services. Furthermore, considering the significant correlation between missed nursing care and job burnout and its dimensions, it is recommended that healthcare policymakers and decision-makers implement strategies to reduce nurse burnout and promote continuous improvement in nursing care and service quality. Continuous training programs focused on burnout reduction skills should be provided to nurses to enhance both nursing care and the overall quality of healthcare services.

## Data Availability

All information and data are available in this article.

## Personal thanks

We extend our sincere gratitude to Kurdistan University of Medical Sciences for their invaluable support in facilitating this study, which was conducted as part of a student thesis. Our heartfelt thanks also go to the dedicated research team, as well as the esteemed faculty and administrators of Kowsar, Towhid, and Besat Teaching Hospitals, affiliated with Kurdistan University of Medical Sciences. Most importantly, we express our deep appreciation to the nurses who participated in this study for their essential cooperation and contribution, without which this research would not have been possible.
